# 4-[2-(2,2-Dimethyl-4,6-dioxo-1,3-dioxan-5-yl­idene)hydrazin-1-yl]benzo­nitrile

**DOI:** 10.1107/S1600536810024025

**Published:** 2010-06-26

**Authors:** Naki Çolak, Yılmaz Yıldırır, Barış Tercan, Emel Ermiş, Tuncer Hökelek

**Affiliations:** a Department of Chemistry, Hitit University, 19030 Ulukavak, Çorum, Turkey; b Department of Chemistry, Gazi University, 06500 Teknikokullar, Ankara, Turkey; c Department of Physics, Karabük University, 78050 Karabük, Turkey; d Department of Chemistry, Faculty of Science, Anadolu University, 26470 Yenibağlar, Eskişehir, Turkey; e Department of Physics, Hacettepe University, 06800 Beytepe, Ankara, Turkey

## Abstract

In the title compound, C_13_H_11_N_3_O_4_, the dioxane ring adopts an envelope conformation with the C atom bonded to the dimethyl group in the flap position [deviation = 0.613 (1) Å]. The nitrile group and the attached benzene ring are roughly coplanar [maximum deviation = 0.087 (1) Å]. An intra­molecular N—H⋯O hydrogen bond involving the hydrazinyl group generates an *S*(6) ring. The N—N and C—N bond lengths indicate that the compound may be a mixture of the azo and hydrazone tautomeric forms but the presence of the N-bound H atom supports the hydrazone form. The crystal structure is stabilized by weak inter­molecular C—H⋯O, C—H⋯N and C—H⋯π inter­actions.

## Related literature

For the applications of related azo compounds, see: Branger *et al.* (1997 ▶); Buchel *et al.* (1995 ▶); Gale *et al.* (1998 ▶); Ikeda & Tsutsumi (1995 ▶); Kang *et al.* (2000 ▶); Karcı *et al.* (2004 ▶); Kim *et al.* (1995 ▶); Kobrakov *et al.* (2004 ▶); Natansohn *et al.* (1992 ▶); Rochon *et al.* (1995 ▶). For related hydrazone structures, see: Çolak *et al.* (2010 ▶); Pavlovic *et al.* (2009 ▶); Seferoğlu *et al.* (2008 ▶); Seferoğlu *et al.* (2009 ▶); Wojciechowski & Szymezak (2007 ▶).
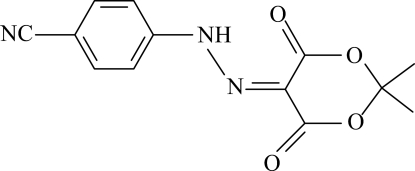


## Experimental

### 

#### Crystal data

C_13_H_11_N_3_O_4_
*M* *_r_* = 273.25
Monoclinic, 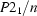

*a* = 9.7617 (2) Å
*b* = 11.0023 (2) Å
*c* = 11.4753 (3) Å
β = 93.796 (1)°
*V* = 1229.76 (5) Å^3^
*Z* = 4
Mo *K*α radiation
μ = 0.11 mm^−1^
*T* = 100 K
0.46 × 0.43 × 0.29 mm

#### Data collection

Bruker Kappa APEXII CCD area-detector diffractometer
Absorption correction: multi-scan (*SADABS*; Bruker, 2005 ▶) *T*_min_ = 0.950, *T*_max_ = 0.968
11746 measured reflections
3098 independent reflections
2591 reflections with *I* > 2σ(*I*)
*R*_int_ = 0.029

#### Refinement

*R*[*F* ^2^ > 2σ(*F* ^2^)] = 0.038
*wR*(*F* ^2^) = 0.108
*S* = 1.04
3098 reflections
225 parameters
All H-atom parameters refined
Δρ_max_ = 0.38 e Å^−3^
Δρ_min_ = −0.24 e Å^−3^

### 

Data collection: *APEX2* (Bruker, 2007 ▶); cell refinement: *SAINT* (Bruker, 2007 ▶); data reduction: *SAINT* program(s) used to solve structure: *SHELXS97* (Sheldrick, 2008 ▶); program(s) used to refine structure: *SHELXL97* (Sheldrick, 2008 ▶); molecular graphics: *ORTEP-3 for Windows* (Farrugia, 1997 ▶); software used to prepare material for publication: *WinGX* (Farrugia, 1999 ▶) and *PLATON* (Spek, 2009 ▶).

## Supplementary Material

Crystal structure: contains datablocks I, global. DOI: 10.1107/S1600536810024025/ci5104sup1.cif

Structure factors: contains datablocks I. DOI: 10.1107/S1600536810024025/ci5104Isup2.hkl

Additional supplementary materials:  crystallographic information; 3D view; checkCIF report

## Figures and Tables

**Table 1:** Hydrogen-bond geometry (Å, °) *Cg*1 is the centroid of the C5–C10 ring.

*D*—H⋯*A*	*D*—H	H⋯*A*	*D*⋯*A*	*D*—H⋯*A*
N2—H2⋯O3	0.92 (2)	1.958 (16)	2.6674 (13)	132 (1)
C9—H9⋯N1^i^	0.94 (2)	2.624 (15)	3.5320 (16)	164 (1)
C10—H10⋯N3^ii^	0.99 (2)	2.485 (15)	3.3876 (16)	152 (1)
C12—H123⋯O2^iii^	0.97 (2)	2.527 (17)	3.4454 (15)	159 (1)
C12—H122⋯*Cg*1^iv^	0.98 (2)	2.491 (15)	3.4575 (13)	171 (1)

Symmetry codes: (i) 
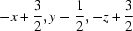
; (ii) 
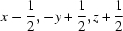
; (iii) 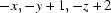; (iv) 
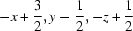
.

**Fig. 1.**
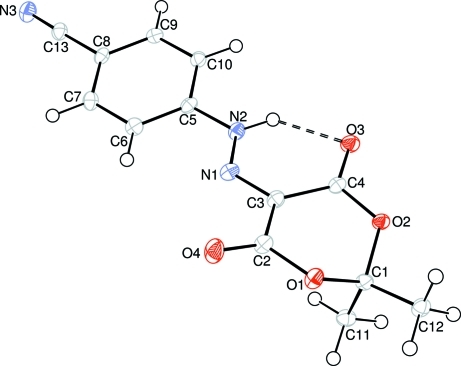
The molecular structure of the title compound, with the atom-numbering scheme. Displacement ellipsoids are drawn at the 50% probability level. The dashed line indicates a hydrogen bond.

## supplementary crystallographic information

### Comment

It has been known for many years that the azo compounds are widely used class of dyes due to their applications in various fields such as the dyeing of textile fibers, the coloring of different materials, colored plastics and electrochemical sensors (Kobrakov *et al.*, 2004; Karcı *et al.*, 2004; Gale *et al.*, 1998). Azo dyes have been attracting intensive interest for their potential use in optical data storage (Natansohn *et al.*, 1992), optical switching (Ikeda & Tsutsumi, 1995), polarization holography (Kim *et al.*, 1995; Rochon *et al.*, 1995), optical modulation (Buchel *et al.*, 1995), nonlinear optics (Branger *et al.*, 1997) and photolabile surfactants (Kang *et al.*, 2000).

The dyes may exist in two possible tautomeric forms, namely azo form and hydrazone form. The azo-hydrazone tautomerism is quite interesting from a theoretical and practical point of view because the two tautomers have different properties. Azo dyes are known to exist in the azo-hydrazone tautomeric forms (Çolak *et al.*, 2010; Pavlovic *et al.*, 2009; Seferoğlu *et al.*, 2009; Seferoğlu *et al.*, 2008; Wojciechowski & Szymezak, 2007). We herein report the crystal structure of the title compound, (I).

The title compound, (I), contains benzonitrile and 2,2-dimethyl-1,3-dioxane -4,6-dione groups connected *via* a hydrazinyl group (Fig. 1). In (I), N1—N2 [1.3082 (14) Å] bond length is between the N═N double bond [1.20–1.28 Å for a real azo compound] and N—N single bond [longer than 1.4 Å] lengths. The N1—C3 and N2—C5 bond lengths are 1.3116 (14) and 1.4075 (14) Å, respectively. The carbonyl O atoms O3 and O4 slightly deviate from the N1/C2–C4 plane by 0.255 (2) and 0.259 (2) Å, respectively. So, the title compound may exist both in azo and hydrazone tautomeric forms, and is mainly in the hydrazone tautomeric form. The C8—C13 [1.4418 (15) Å] bond length is longer for a C(sp^2^)—C(sp^1^) bond, but in agreement with the previously reported value [1.442 (3) Å; Çolak *et al.*, 2010).

An intramolecular N2—H2···O3 hydrogen bond (Table 1) results in the formation of a nearly planar [with a maximum deviation of 0.056 (1) Å for atom C4] six-membered ring C (O3/N1/N2/H2/C3/C4), which is oriented with respect to the benzonitrile ring B (C5–C10) at a dihedral angle of 7.8 (43)°. Atoms N1, N2, N3 and C13 are displaced by -0.162 (2), 0.038 (2), 0.049 (2) and 0.028 (2) Å from the plane of ring B, respectively. The benzonitrile and hydrazinyl groups (4-hydrazinylbenzonitrile) are essentially coplanar [with a maximum deviation of -0.087 (1) Å for atom N1]. The dioxane ring A (O1/O2/C1–C4) is not planar having envelope conformation with atom C1 displaced by 0.613 (1) Å from the plane of the other ring atoms.

In the crystal structure, weak C—H···O and C—H···N hydrogen bonds (Table 1) may be effective in the stabilization of the crystal packing. There also exists a weak C—H···π interaction (Table 1).

### Experimental

A hydrochloric acid solution (2.5 ml) of 4-aminobenzonitrile (1.18 g, 10 mmol) and an aqueous solution (10 ml) of sodium nitrite (0.69 g, 10 mmol) were mixed and stirred at 273 K for 1 h. To this solution, an ethanol solution (10 ml) of the coupling component 2,2-dimethyl-1,3-dioxane-4,6-dione (1.44 g, 10 mmol) was added and the stirring was continued at 273 K for 4 h. The resulting product was filtered and washed with water, dried and crystallized from ethanol (yield 1.88 g, 69%; m.p. 440–442 K).

### Refinement

H atoms were located in difference Fourier maps and refined isotropically.

### Crystal data

**Table:**

C_13_H_11_N_3_O_4_	*F*(000) = 568
*M**_r_* = 273.25	*D*_x_ = 1.476 Mg m^−^^3^
Monoclinic, *P*2_1_/*n*	Mo *K*α radiation, λ = 0.71073 Å
Hall symbol: -P 2yn	Cell parameters from 4103 reflections
*a* = 9.7617 (2) Å	θ = 2.6–28.4°
*b* = 11.0023 (2) Å	µ = 0.11 mm^−^^1^
*c* = 11.4753 (3) Å	*T* = 100 K
β = 93.796 (1)°	Block, yellow
*V* = 1229.76 (5) Å^3^	0.46 × 0.43 × 0.29 mm
*Z* = 4	

### Data collection

**Table:**

Bruker Kappa APEXII CCD area-detector diffractometer	3098 independent reflections
Radiation source: fine-focus sealed tube	2591 reflections with *I* > 2σ(*I*)
graphite	*R*_int_ = 0.029
φ and ω scans	θ_max_ = 28.5°, θ_min_ = 2.6°
Absorption correction: multi-scan (*SADABS*; Bruker, 2005)	*h* = −13→13
*T*_min_ = 0.950, *T*_max_ = 0.968	*k* = −14→14
11746 measured reflections	*l* = −15→8

### Refinement

**Table:**

Refinement on *F*^2^	Primary atom site location: structure-invariant direct methods
Least-squares matrix: full	Secondary atom site location: difference Fourier map
*R*[*F*^2^ > 2σ(*F*^2^)] = 0.038	Hydrogen site location: inferred from neighbouring sites
*wR*(*F*^2^) = 0.108	All H-atom parameters refined
*S* = 1.04	*w* = 1/[σ^2^(*F*_o_^2^) + (0.0626*P*)^2^ + 0.2729*P*] where *P* = (*F*_o_^2^ + 2*F*_c_^2^)/3
3098 reflections	(Δ/σ)_max_ = 0.001
225 parameters	Δρ_max_ = 0.38 e Å^−^^3^
0 restraints	Δρ_min_ = −0.24 e Å^−^^3^

### Special details

**Table:**

Geometry. All e.s.d.'s (except the e.s.d. in the dihedral angle between two l.s. planes) are estimated using the full covariance matrix. The cell e.s.d.'s are taken into account individually in the estimation of e.s.d.'s in distances, angles and torsion angles; correlations between e.s.d.'s in cell parameters are only used when they are defined by crystal symmetry. An approximate (isotropic) treatment of cell e.s.d.'s is used for estimating e.s.d.'s involving l.s. planes.
Refinement. Refinement of *F*^2^ against ALL reflections. The weighted *R*-factor *wR* and goodness of fit *S* are based on *F*^2^, conventional *R*-factors *R* are based on *F*, with *F* set to zero for negative *F*^2^. The threshold expression of *F*^2^ > σ(*F*^2^) is used only for calculating *R*-factors(gt) *etc*. and is not relevant to the choice of reflections for refinement. *R*-factors based on *F*^2^ are statistically about twice as large as those based on *F*, and *R*- factors based on ALL data will be even larger.

### Fractional atomic coordinates and isotropic or equivalent isotropic displacement parameters (Å^2^)

**Table:**

	*x*	*y*	*z*	*U*_iso_*/*U*_eq_	
O1	0.16363 (8)	0.74524 (7)	0.85585 (7)	0.0172 (2)	
O2	0.17892 (8)	0.56739 (7)	0.97179 (7)	0.01470 (19)	
O3	0.35656 (9)	0.44276 (8)	0.96914 (7)	0.0187 (2)	
O4	0.32521 (9)	0.79574 (8)	0.73794 (8)	0.0249 (2)	
N1	0.44403 (10)	0.56888 (9)	0.76510 (9)	0.0165 (2)	
N2	0.51381 (10)	0.46953 (9)	0.78943 (9)	0.0159 (2)	
H2	0.4942 (16)	0.4243 (15)	0.8538 (15)	0.028 (4)*	
N3	1.03756 (11)	0.29578 (10)	0.47015 (10)	0.0232 (2)	
C1	0.08704 (11)	0.64589 (10)	0.90176 (10)	0.0143 (2)	
C2	0.27845 (12)	0.71950 (11)	0.79881 (10)	0.0172 (2)	
C3	0.34022 (12)	0.59901 (10)	0.82608 (10)	0.0155 (2)	
C4	0.29477 (11)	0.52812 (10)	0.92521 (10)	0.0144 (2)	
C5	0.62374 (11)	0.43844 (10)	0.72180 (10)	0.0148 (2)	
C6	0.64458 (13)	0.49922 (11)	0.61746 (10)	0.0173 (2)	
H6	0.5852 (15)	0.5636 (14)	0.5904 (13)	0.020 (4)*	
C7	0.75216 (13)	0.46401 (11)	0.55177 (10)	0.0175 (2)	
H7	0.7668 (15)	0.5059 (14)	0.4788 (13)	0.024 (4)*	
C8	0.83788 (12)	0.36875 (11)	0.59035 (10)	0.0155 (2)	
C9	0.81720 (12)	0.30898 (11)	0.69557 (10)	0.0161 (2)	
H9	0.8752 (14)	0.2457 (14)	0.7224 (12)	0.020 (4)*	
C10	0.70992 (12)	0.34407 (11)	0.76131 (10)	0.0159 (2)	
H10	0.6926 (14)	0.3023 (14)	0.8350 (13)	0.021 (4)*	
C11	0.01442 (13)	0.57371 (11)	0.80405 (10)	0.0178 (2)	
H111	0.0795 (15)	0.5338 (14)	0.7556 (13)	0.022 (4)*	
H112	−0.0444 (15)	0.5101 (14)	0.8393 (13)	0.023 (4)*	
H113	−0.0456 (15)	0.6270 (15)	0.7544 (14)	0.026 (4)*	
C12	−0.00717 (13)	0.70098 (11)	0.98606 (10)	0.0169 (2)	
H121	0.0494 (16)	0.7364 (15)	1.0534 (13)	0.026 (4)*	
H122	−0.0616 (15)	0.7636 (14)	0.9446 (13)	0.021 (4)*	
H123	−0.0673 (16)	0.6388 (16)	1.0133 (15)	0.035 (4)*	
C13	0.94875 (12)	0.32918 (11)	0.52248 (10)	0.0177 (2)	

### Atomic displacement parameters (Å^2^)

**Table:**

	*U*^11^	*U*^22^	*U*^33^	*U*^12^	*U*^13^	*U*^23^
O1	0.0168 (4)	0.0111 (4)	0.0244 (4)	0.0003 (3)	0.0057 (3)	0.0024 (3)
O2	0.0132 (4)	0.0139 (4)	0.0169 (4)	0.0027 (3)	0.0012 (3)	0.0015 (3)
O3	0.0164 (4)	0.0178 (4)	0.0220 (4)	0.0033 (3)	0.0011 (3)	0.0027 (3)
O4	0.0244 (5)	0.0191 (5)	0.0321 (5)	0.0004 (4)	0.0096 (4)	0.0083 (4)
N1	0.0144 (5)	0.0146 (5)	0.0204 (5)	−0.0002 (4)	0.0009 (4)	−0.0023 (4)
N2	0.0151 (5)	0.0149 (5)	0.0179 (5)	0.0004 (4)	0.0031 (4)	−0.0005 (4)
N3	0.0227 (6)	0.0233 (6)	0.0244 (5)	0.0003 (4)	0.0076 (4)	0.0001 (4)
C1	0.0135 (5)	0.0111 (5)	0.0182 (5)	0.0005 (4)	0.0010 (4)	0.0024 (4)
C2	0.0152 (6)	0.0159 (6)	0.0208 (5)	−0.0001 (4)	0.0025 (4)	−0.0003 (4)
C3	0.0143 (5)	0.0140 (5)	0.0184 (5)	−0.0005 (4)	0.0017 (4)	−0.0006 (4)
C4	0.0127 (5)	0.0135 (5)	0.0170 (5)	−0.0010 (4)	0.0008 (4)	−0.0027 (4)
C5	0.0119 (5)	0.0143 (5)	0.0182 (5)	−0.0024 (4)	0.0014 (4)	−0.0037 (4)
C6	0.0178 (6)	0.0137 (5)	0.0205 (5)	−0.0001 (4)	0.0014 (4)	0.0001 (4)
C7	0.0196 (6)	0.0155 (5)	0.0178 (5)	−0.0026 (4)	0.0039 (4)	0.0004 (4)
C8	0.0134 (5)	0.0155 (5)	0.0179 (5)	−0.0031 (4)	0.0028 (4)	−0.0022 (4)
C9	0.0137 (5)	0.0160 (5)	0.0185 (5)	0.0003 (4)	0.0001 (4)	−0.0002 (4)
C10	0.0144 (5)	0.0177 (5)	0.0155 (5)	−0.0026 (4)	0.0010 (4)	−0.0004 (4)
C11	0.0174 (6)	0.0170 (6)	0.0188 (5)	0.0019 (5)	−0.0009 (4)	−0.0015 (4)
C12	0.0162 (6)	0.0141 (5)	0.0207 (5)	0.0032 (4)	0.0037 (4)	0.0007 (4)
C13	0.0178 (6)	0.0167 (6)	0.0188 (5)	−0.0029 (4)	0.0024 (4)	0.0006 (4)

### Geometric parameters (Å, °)

**Table:**

O1—C1	1.4435 (13)	C6—C7	1.3878 (16)
O1—C2	1.3645 (13)	C6—H6	0.954 (15)
O2—C1	1.4493 (13)	C7—C8	1.3949 (17)
O2—C4	1.3533 (13)	C7—H7	0.974 (15)
O3—C4	1.2081 (14)	C8—C9	1.4012 (16)
O4—C2	1.2009 (14)	C8—C13	1.4418 (15)
N1—N2	1.3082 (14)	C9—C10	1.3854 (15)
N1—C3	1.3116 (14)	C9—H9	0.936 (15)
N2—C5	1.4075 (14)	C10—H10	0.986 (15)
N2—H2	0.922 (17)	C11—C1	1.5117 (16)
N3—C13	1.1473 (15)	C11—H111	0.976 (15)
C1—C12	1.5055 (15)	C11—H113	0.984 (16)
C2—C3	1.4812 (16)	C11—H112	1.007 (16)
C3—C4	1.4718 (15)	C12—H122	0.975 (15)
C5—C6	1.3981 (16)	C12—H121	0.999 (16)
C5—C10	1.3932 (17)	C12—H123	0.967 (17)
			
C2—O1—C1	118.66 (9)	C7—C6—H6	119.7 (9)
C4—O2—C1	118.29 (8)	C6—C7—C8	119.80 (11)
N2—N1—C3	120.47 (10)	C6—C7—H7	119.6 (9)
N1—N2—C5	119.38 (10)	C8—C7—H7	120.6 (9)
N1—N2—H2	119.3 (10)	C7—C8—C9	120.56 (10)
C5—N2—H2	121.1 (10)	C7—C8—C13	120.76 (10)
O1—C1—O2	109.71 (9)	C9—C8—C13	118.68 (11)
O1—C1—C11	110.85 (9)	C8—C9—H9	120.9 (9)
O1—C1—C12	106.43 (9)	C10—C9—C8	119.72 (11)
O2—C1—C11	109.92 (9)	C10—C9—H9	119.4 (9)
O2—C1—C12	105.40 (9)	C5—C10—H10	119.5 (9)
C12—C1—C11	114.31 (10)	C9—C10—C5	119.50 (10)
O1—C2—C3	114.85 (10)	C9—C10—H10	121.0 (9)
O4—C2—O1	119.33 (11)	C1—C11—H111	111.6 (9)
O4—C2—C3	125.66 (11)	C1—C11—H113	110.4 (9)
N1—C3—C2	115.57 (10)	C1—C11—H112	108.6 (9)
N1—C3—C4	124.16 (11)	H111—C11—H112	109.0 (12)
C4—C3—C2	119.88 (10)	H111—C11—H113	108.9 (13)
O2—C4—C3	116.02 (10)	H113—C11—H112	108.3 (12)
O3—C4—O2	119.38 (10)	C1—C12—H121	108.9 (9)
O3—C4—C3	124.52 (10)	C1—C12—H122	107.9 (8)
C6—C5—N2	121.03 (11)	C1—C12—H123	109.4 (10)
C10—C5—N2	117.89 (10)	H121—C12—H123	110.0 (13)
C10—C5—C6	121.08 (10)	H122—C12—H121	110.9 (13)
C5—C6—H6	120.9 (9)	H122—C12—H123	109.6 (13)
C7—C6—C5	119.34 (11)	N3—C13—C8	178.56 (13)
			
C2—O1—C1—O2	51.23 (12)	O4—C2—C3—N1	−9.81 (19)
C2—O1—C1—C11	−70.35 (13)	O4—C2—C3—C4	163.35 (12)
C2—O1—C1—C12	164.79 (10)	N1—C3—C4—O2	−174.73 (10)
C1—O1—C2—O4	163.20 (11)	N1—C3—C4—O3	8.59 (19)
C1—O1—C2—C3	−21.07 (14)	C2—C3—C4—O2	12.73 (16)
C4—O2—C1—O1	−50.36 (12)	C2—C3—C4—O3	−163.95 (11)
C4—O2—C1—C12	−164.59 (9)	N2—C5—C6—C7	−178.47 (10)
C4—O2—C1—C11	71.77 (12)	C10—C5—C6—C7	0.62 (18)
C1—O2—C4—O3	−163.46 (10)	N2—C5—C10—C9	178.44 (10)
C1—O2—C4—C3	19.68 (14)	C6—C5—C10—C9	−0.67 (18)
C3—N1—N2—C5	179.24 (10)	C5—C6—C7—C8	0.05 (18)
N2—N1—C3—C2	173.80 (10)	C6—C7—C8—C9	−0.65 (18)
N2—N1—C3—C4	0.97 (18)	C6—C7—C8—C13	178.87 (11)
N1—N2—C5—C6	−11.37 (17)	C7—C8—C9—C10	0.60 (18)
N1—N2—C5—C10	169.52 (10)	C13—C8—C9—C10	−178.93 (11)
O1—C2—C3—N1	174.77 (10)	C8—C9—C10—C5	0.06 (17)
O1—C2—C3—C4	−12.07 (16)		

### Hydrogen-bond geometry (Å, °)

**Table:**

Cg1 is the centroid of the C5–C10 ring.

**Table:**

*D*—H···*A*	*D*—H	H···*A*	*D*···*A*	*D*—H···*A*
N2—H2···O3	0.92 (2)	1.958 (16)	2.6674 (13)	132 (1)
C9—H9···N1^i^	0.94 (2)	2.624 (15)	3.5320 (16)	164 (1)
C10—H10···N3^ii^	0.99 (2)	2.485 (15)	3.3876 (16)	152 (1)
C12—H123···O2^iii^	0.97 (2)	2.527 (17)	3.4454 (15)	159 (1)
C12—H122···Cg1^iv^	0.98 (2)	2.491 (15)	3.4575 (13)	171 (1)

Symmetry codes: (i) −*x*+3/2, *y*−1/2, −*z*+3/2; (ii) *x*−1/2, −*y*+1/2, *z*+1/2; (iii) −*x*, −*y*+1, −*z*+2; (iv) −*x*+3/2, *y*−1/2, −*z*+1/2.
